# Detection of an *en masse* and reversible B- to A-DNA conformational transition in prokaryotes in response to desiccation

**DOI:** 10.1098/rsif.2014.0454

**Published:** 2014-08-06

**Authors:** Donna R. Whelan, Thomas J. Hiscox, Julian I. Rood, Keith R. Bambery, Don McNaughton, Bayden R. Wood

**Affiliations:** 1 Centre for Biospectroscopy, School of Chemistry, Monash University, Victoria 3800, Australia; 2 Department of Microbiology, School of Biomedical Sciences, Monash University, Victoria 3800, Australia; 3 Australian Synchrotron, 800 Blackburn Road, Clayton, Victoria 3168, Australia

**Keywords:** DNA conformation, attenuated total reflection–Fourier transform infrared spectroscopy, bacteria, A-DNA, infrared

## Abstract

The role that DNA conformation plays in the biochemistry of cells has been the subject of intensive research since DNA polymorphism was discovered. B-DNA has long been considered the native form of DNA in cells although alternative conformations of DNA are thought to occur transiently and along short tracts. Here, we report the first direct observation of a fully reversible *en masse* conformational transition between B- and A-DNA within live bacterial cells using Fourier transform infrared (FTIR) spectroscopy. This biospectroscopic technique allows for non-invasive and reagent-free examination of the holistic biochemistry of samples. For this reason, we have been able to observe the previously unknown conformational transition in all four species of bacteria investigated. Detection of this transition is evidence of a previously unexplored biological significance for A-DNA and highlights the need for new research into the role that A-DNA plays as a cellular defence mechanism and in stabilizing the DNA conformation. Such studies are pivotal in understanding the role of A-DNA in the evolutionary pathway of nucleic acids. Furthermore, this discovery demonstrates the exquisite capabilities of FTIR spectroscopy and opens the door for further investigations of cell biochemistry with this under-used technique.

## 1. Introduction

The molecular conformation of DNA *in situ* has been a point of interest ever since Watson and Crick's X-ray diffraction studies and the ensuing analyses revealed crystal polymorphism of the double helix [1–5]. Not only did they solve the crystal structure of the assumed native, fully hydrated B-DNA, they also detected and identified a more dehydrated A-DNA polymorph in DNA crystals. As this polymorphism was detected over 60 years ago, dozens of alternative double-stranded conformations have been identified including Z-DNA [6] and Holliday junctions [7], as well as triplex [8,9] and quadruplex conformations [10,11]. The biological significance of these non-canonical conformations, however, has been hotly contested because of a lack of techniques capable of determining *in situ* DNA conformation, resulting in a heavy reliance on indirect methods such as crystallography [12,13], computational chemistry [14,15] and electron microscopy [16,17].

Nonetheless, several DNA conformations are now believed to be present in cells and to be involved in biologically relevant reactions [16]. Of the conformations now established as biologically relevant, A-DNA has been the most researched and has been identified as the most biologically active non-B-DNA conformation. In the last two decades, sequences containing 5′-CC-3′ and 5′-ACT-3′ repeats have been demonstrated to be predisposed to assuming an A-DNA form most easily [18]. Furthermore, the transition to A-DNA is required for many different transient interactions with enzymes [12,19,20], counter-ions [13] and ligands [21]. A-DNA has also been strongly implicated through protein–DNA crystal structures as a necessary structure in several polymerase reactions [22] and in all RNA/RNA [23,24] and hybrid helices [25]. In a handful of studies, A-DNA has also been demonstrated to have increased stability in response to UV radiation [26,27], chemical toxicity [28] and desiccation [29,30], though the significance of this finding has been questioned owing to the prevalence of B-DNA in live cells.

One method capable of determining DNA conformation is infrared (IR) spectroscopy and, following the discovery of the double helix, the conformation-specific IR absorptions of both B- and A-DNA were exhaustively catalogued and used to complement crystallography data [20,31–33]. In more recent years, Fourier transform infrared (FTIR) spectroscopy has been applied to intact cell and tissue samples as one of several techniques known collectively as biospectroscopy. These methods have been highlighted as both biochemical and medical diagnostic tools capable of detecting disease state [34], cell cycle [35], the onset of apoptosis and necrosis [36], as well as for investigating cell interactions with drugs, chemicals and pathogens [37]. This is because FTIR spectroscopy provides a holistic snapshot of the biochemical make-up of a sample based on the vibrational absorption of all the sample constituents. Different chemical bonds absorb at different energies and the degree of absorption is directly proportional to concentration. FTIR spectroscopy can thus be applied to live samples without any requirement for staining or targeting and with no danger of perturbation to the sample itself.

However, interpretation of the FTIR spectra of biological samples is often rendered difficult owing to the complicated overlap of absorptions from so many different molecules. Accordingly, band assignments are typically made to a set of biomolecules such as lipids, proteins or nucleic acids (see the electronic supplementary material, figure S1) [38]. Moreover, intense water absorptions in the biochemically relevant area of the IR spectrum (4000–600 cm^−1^) continue to necessitate the use of a synchrotron source and small path lengths for sensitive measurements of hydrated, live cells because of the high throughput required to transmit light through aqueous media, the cell and IR transparent windows [39]. Over the past decade, further complications in the successful application of FTIR spectroscopy to biological samples have stalled the uptake of this technique. Cells were found to cause Mie scattering and were susceptible to refractive index mismatching, both of which manifested as nonlinear baselines and shifting absorption peaks and intensities and which led to incorrect band assignments [40]. However, recent advances in methodologies along with a better understanding of these effects have led to the development of correctional algorithms and, in the process, demonstrated the true potential of FTIR spectroscopy for the analysis of biological specimens [41–43].

Using these advances, we recently reported live-cell synchrotron–FTIR spectroscopic detection of predominantly B-DNA in eukaryotic cells [35,44]. In that study, an *en masse* transition of the genomic DNA from B-DNA to an A-like DNA form was observed during dehydration in all eukaryotic cells examined. Importantly, the full reversal of the B to A transition was also detected upon rehydration [44].

This transition of the cellular B- to A-DNA upon dehydration had previously only been reported once [29]. Crystallographic work by Mohr *et al*. identified the same reversible transition of the DNA in *Bacillus subtilis* upon natural desiccation. In Mohr *et al*.'s [29] research, the transition to A-DNA was first reported as an effect of the binding of DNA-protecting proteins to the genome during desiccation of the spores. In a second publication, the A-DNA conformation was identified as the primary mode of resistance to DNA damage, with the DNA-protecting proteins providing the mechanism by which the transition was achieved [30].

This result, in combination with other reports of A-DNA having increased resistance to various forms of damage and with the spectroscopic evidence for the surprisingly reversible B- to A-DNA transition in eukaryotic cells, highlights the potential biological significance of the genome-wide B- to A-DNA transition. Furthermore, FTIR spectroscopy has previously detected conformational change in the DNA of cells in tissue sections via the symmetric phosphate vibration [45]. In this research, conformational differences as detected using FTIR spectroscopy were observed to be strongly prescriptive of stem cell regions. This demonstrates the ability of this technique to detect otherwise undiscoverable DNA conformational changes.

Although our previous research did not endeavour to maintain cell viability during dehydration and detection of the conformational transition [44], here we report on the detection of the B–A–B-DNA transition in desiccation-resistant bacteria with no known mechanism for DNA stabilization. The bacteria were rehydrated from a dormant state with no loss of functionality, demonstrating the B- to A-DNA transition in a functional biological system.

## 2. Results

**Figure 1.**
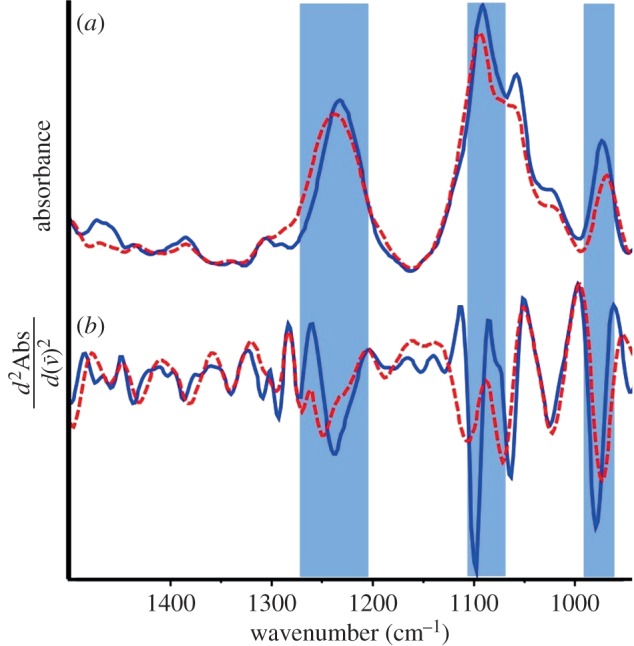
FTIR spectra (*a*) and calculated second derivative spectra (*b*) of double-stranded DNA in the dehydrated A-DNA conformation (dashed red) and after rehydration in the B-DNA conformation (solid blue). Shading indicates, from left to right, the anti-symmetric phosphate stretch, the symmetric phosphate stretch and the backbone carbon–carbon vibration. (Online version in colour.)

Figure 1 shows the raw and second derivative spectra of partially dehydrated A-DNA (red) and the same sample after rehydration to B-DNA (blue). In the backbone fingerprint region of A-DNA, the most prominent bands are observed to be the anti-symmetric phosphate stretching vibration (*v*_asym_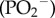 = 1237 cm^−1^), the symmetric phosphate stretching vibration (*v*_sym_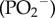 = 1088 cm^−1^) and the backbone carbon–carbon vibration (*v*(C–C) = 966 cm^−1^). Upon rehydration to B-DNA, these bands all undergo significant observable changes. The *v*_asym_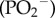 red-shifts by 14 cm^−1^ to 1223 cm^−1^, the *v*(C–C) blue shifts to 969 cm^−1^ and the *v*_sym_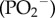 decreases in intensity. These shifts have previously been associated with the respective A- and B-conformations of DNA and used to monitor polymorphism [33]. Moreover, we recently demonstrated that the change in intensity of the *v*_sym_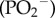 was due to a change in molar extinction coefficient, also related to the B–A transition [43]. Figure S1 in the electronic supplementary material highlights the various peaks in FTIR spectra of bacteria attributed to protein, carbohydrate, lipid and nucleic acid absorptions, as well as the high absorbance of water vibrations in the approximately 3300 cm^−1^ and approximately 1625 cm^−1^ regions. Because of this wide distribution of absorbances and the interference from the water absorptions, we focused mainly on the phosphodiester backbone region (1300–950 cm^−1^).

**Figure 2.**
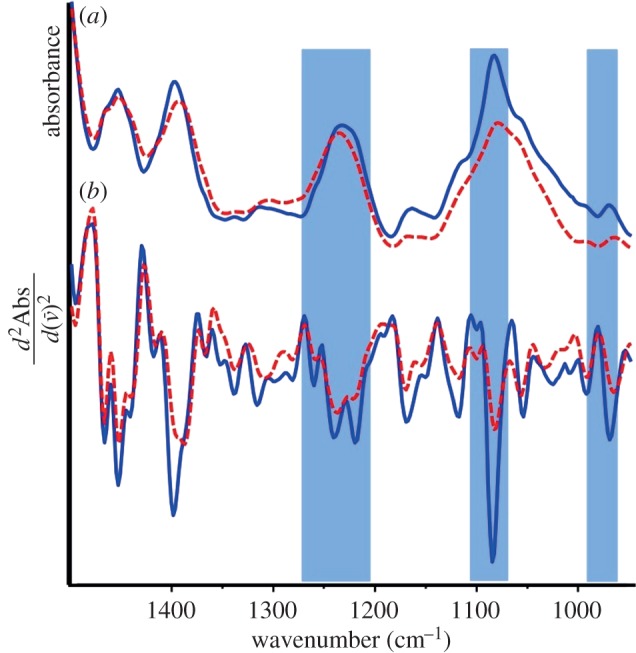
FTIR spectra (*a*) and calculated second derivatives (*b*) of *P. vulgaris* in the dormant dehydrated state (dashed red) and after rehydration (solid blue). From left to right, shading highlights the characteristic anti-symmetric phosphate stretch, the symmetric phosphate stretch and the backbone carbon–carbon vibration that are diagnostic of the A- to B-DNA transition. (Online version in colour.)

The spectra of a dehydrated and subsequently rehydrated viable *Proteus vulgaris* culture along with the calculated second derivative spectra over the region 1300–950 cm^−1^ are shown in figure 2. By examining the second derivative spectra overlapping absorptions can be better resolved and hence changes in relative absorption intensity and position can be detected more clearly. The IR spectrum of the dehydrated, dormant culture demonstrated the three diagnostic A-DNA absorptions: *v*_asym_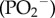 = 1239 cm^−1^, *v*(C–C) = 966 cm^−1^ and the less intense *v*_sym_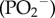 = 1084 cm^−1^. By contrast, the spectrum of the hydrated culture had absorptions indicative of B-DNA: *v*_asym_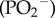 = 1222 cm^−1^, *v*(C–C) = 969 cm^−1^ and the more intense *v*_sym_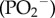 = 1086 cm^−1^. It should be noted that, for the *v*_asym_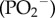 absorption, it is particularly difficult to detect this transition owing to overlapping amide III, phospholipid and phosphorylated protein vibrations [46], all of which are observed in the second derivative. This overlap has been reported before; however, the band at approximately 1238 cm^−1^, which is red-shifted by approximately 14 cm^−1^ to 1224 cm^−1^ is specific for the DNA conformational change and is not from other molecular vibrational modes that appear in the vicinity of this band. Accordingly, in the dormant culture, the A-DNA signal (1239 cm^−1^) is more prominent than the B-DNA signal (1222 cm^−1^). The red-shift of the *v*_sym_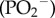 and broadening of the *v*(C–C) have similarly been observed previously in cell spectra and are thought to be due to overlapping absorptions from other biomolecules as well as band-broadening due to steric crowding. These spectra are representative of repeated experiments using the *P. vulgaris* strain along with a second cycle of the rehydrated culture through a dehydration cycle.

**Figure 3.**
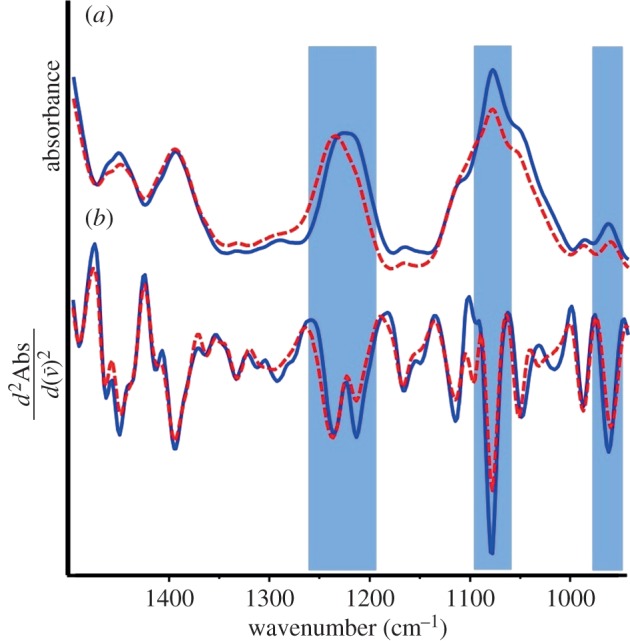
FTIR spectra (*a*) and calculated second derivatives (*b*) of *E. coli* in the dormant dehydrated state (dashed red) and after rehydration (solid blue). From left to right, shading highlights the characteristic anti-symmetric phosphate stretch, the symmetric phosphate stretch and the backbone carbon–carbon vibration that are diagnostic of the A- to B-DNA transition. (Online version in colour.)

*Escherichia coli* spectra also demonstrated A- and B-DNA absorptions with representative spectra of the dehydrated and rehydrated culture shown in figure 3. Again the *v*_asym_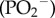 was observed to be split into two absorptions in the second derivative spectra of rehydrated samples, with the dehydrated culture exhibiting a more intense peak at 1241 cm^−1^ indicative of A-DNA and the rehydrated bacteria having a significantly more intense 1220 cm^−1^ absorption. In the underivatized spectra, the *v*_asym_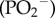 shifts from 1240 cm^−1^ dehydrated to 1232 cm^−1^ when rehydrated in accordance with the A–B transition. Furthermore, the *v*_sym_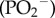 (1085 cm^−1^) increases dramatically in intensity upon rehydration and the *v*(C–C) shifts from 966 to 969 cm^−1^. Following rehydration of *P. vulgaris* and *E. coli*, the number of viable bacteria was determined to be 5.2 × 10^6^ and 8.3 × 10^6^ CFU ml^−1^, respectively.

The spectra of dehydrated and hydrated *Salmonella typhimurium* and *Pseudomonas aeruginosa* cultures showed that the *v*_sym_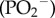 and *v*(C–C) absorptions consistently undergo the typical absorption changes indicative of the A–B transition upon rehydration (electronic supplementary material, figures S2 and S3). The expected absorption changes of the *v*_asym_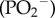 absorption were not observed in the spectra of these two cultures; however, analysis of the second derivative spectra demonstrates the presence of peak splitting here in both the hydrated and dehydrated states that can be attributed to several overlapping non-DNA bands. It seems probable, considering the presence of other backbone A- and B-DNA diagnostic absorptions, that the *v*_asym_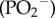 shift was present but was more difficult to unambiguously detect in these species.

**Figure 4.**
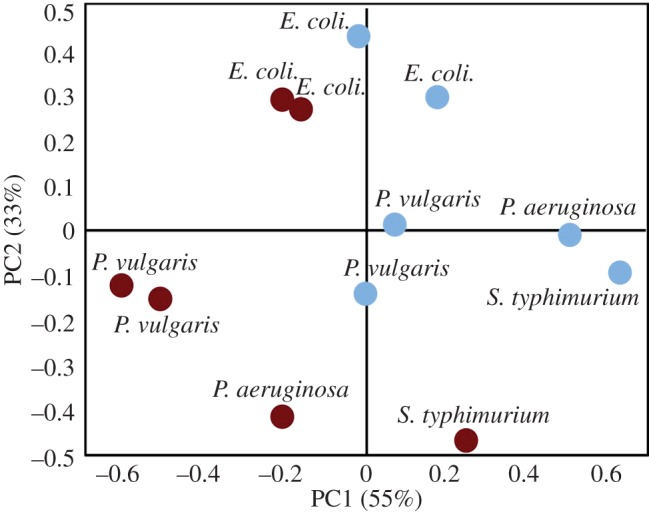
PCA scores plot of 12 FTIR spectra of hydrated (light blue) and dehydrated (dark red) bacteria in principal component 1 and 2 space. (Online version in colour.)

To further confirm the A- to B-DNA conformational change during rehydration, pairs of spectra of the four bacterial strains were used in a principal component analysis (PCA) model. The scores plot of the first two principal components (PC1 and PC2) is shown in figure 4, and the corresponding loadings plots are available in the electronic supplementary material, figure 4S. The PCA was conducted using the entire 1300–950 cm^−1^ phosphodiester region, and all species were successfully separated by hydration state along PC1 except *S. typhimurium*. The spectrum of dehydrated *S. typhimurium* was apparent in highly negative PC2 space and appeared quite separate from the hydrated spectra of the other species; however, it was still found in significantly more negative PC1 space than in the equivalent hydrated spectrum. This trend was observed for all pairs of spectra: the spectra of dehydrated cultures consistently being observed in more negative PC1 space than the spectra of the subsequently rehydrated culture. To confirm that this trend was due to the A- to B-DNA transition, the PC1 loadings plot was examined (electronic supplementary material, figure 4S) and the separation of spectra along PC1 was found to be primarily due to DNA absorptions. In accordance with direct observation of the spectra (figures 2 and 3; electronic supplementary material, figures 2S and 3S), the major source of variance along PC1 was the *v*_sym_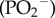 (1084 cm^−1^) with spectra in the positive PC1 space (hydrated) having more intense *v*_sym_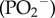 absorptions. The *v*_asym_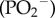 was the only band detected as being more intense in the negative PC1 space, with the absorption showing characteristics of a shift in the PC1 loading. The positive PC1 space was observed to have stronger contributions from the red-shifted B-DNA *v*_asym_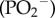 (1210 cm^−1^ in PC1), whereas the negative PC1 space was found to have stronger contributions from the blue-shifted A-DNA *v*_asym_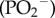 (1255 cm^−1^). The *v*(C–C) is detected at 970 cm^−1^ in the PC1 loadings plot and found to contribute most strongly to the positive PC1 space. This result was in good agreement with the observation of the gain in intensity in this absorption band during rehydration. Owing to this large change in intensity in the raw spectra, the *v*(C–C) shift was not detected.

The separation of hydrated from dehydrated bacteria spectra of all four species along PC1, in particular *P. vulgaris*, *E. coli* and *P. aeruginosa*, which cluster completely separately, demonstrated a common difference in these spectra. Examination of the PC1 loadings plot confirmed that the correlation was indicative of the A- to B-DNA transition.

## 3. Discussion

The perceived biological significance of A-DNA has previously been confined to transient transitions of short stretches of DNA from the native B-form [16]. Here, we show a concerted transition of the DNA of several species of bacteria to B-DNA during rehydration from a dormant, but functional state in which the DNA is in the A-form. This result clearly demonstrates that the DNA of these prokaryotes transitions to the A-form during desiccation and then returns to the native B-form in parallel with regaining full functionality upon rehydration. These observations come from using FTIR spectroscopy, a technique capable of monitoring *in situ* DNA conformation directly and non-invasively [44]. This capability is in contrast to other typical methods for examining DNA conformation such as crystallography and electron microscopy, which both require rigorous sample preparation and cannot be used for live-cell measurement. FTIR spectroscopy, on the other hand, has been shown to be very sensitive to DNA conformation with changes in absorptions that enable differentiation between A- and B-DNA as well as being directly proportional to concentration in the cellular environment [43].

One limitation of FTIR is that it does not have the required sensitivity to detect conformational changes on short tracts of DNA and only *en masse* transitions are possible. However, because DNA typically constitutes less than 5% of bacterial cell dry weight [47], clear elucidation of these changes to the FTIR spectrum relies on a fortuitous lack of overlapping absorptions in any given sample. In an earlier study, we reported the bands diagnostic of the B–A–B transition for several eukaryotic cells [44]. The main bands indicative of the B–A transition in cell spectra include the symmetric and anti-symmetric phosphate vibrations because these bands are not as heavily overlapped by protein absorptions (amide I: approx. 1650 cm^−1^, amide II: 1550 cm^−1^) and because the phosphate moiety is four times more concentrated that the nucleic base moieties.

In both the *E. coli* and *P. vulgaris* samples, all the diagnostic DNA backbone absorption changes were clearly observed, whereas in the *S. typhimurium* and *P. aeruginosa* spectra the band most susceptible to overlapping bands, *v*_asym_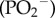, did not show a clear A- to B-DNA transition. PCA, however, does differentiate between hydrated and dehydrated cultures successfully based predominantly on A- and B-DNA absorptions. The lack of clear DNA bands observed in some regions of the bacterial spectra is the result of overlapping bands masking the spectral changes associated with the B–A–B transition.

With this in mind, the unequivocal detection of the A- to B-DNA transition upon rehydration in several different strains of desiccation-resistant bacterial cells as well as in non-functional eukaryotic cell raises questions about the role of the *en masse* transition to A-DNA. Particularly because the A-form of DNA has previously been implicated in several studies as the conformation central to the evolution of nucleic acids [48,49] and in resisting DNA damage. A-DNA has been documented *ex vivo* as having increased resistance to damage by UV [26,27], desiccation [29] and radio-sensitization [28] and is the assumed conformation taken on by *B. subtilis* spores in a concerted protein–DNA response to dehydration [30].

Because of the lack of appropriate methodology, A-DNA has not previously been considered the conformation of DNA prevalent during the desiccation and survival of prokaryotes. Here, we have used FTIR spectroscopy to directly and non-invasively monitor a B–A–B transition of the DNA in four species of bacteria, demonstrating a newly found significance of this conformation and the potential central role of A-DNA in cells under stress. Observation of the predominantly A-form DNA in prokaryotes necessitates further consideration of the B–A conformational transition and its role in defence mechanisms as well as its potential evolutionary role with regard to the ongoing debate over the origins of nucleic acids and their central role in life [50].

## 4. Material and methods

### 4.1. Bacteria culture

*Proteus vulgaris* or *E. coli* were cultured in Luria-Bertani broth at 37°C, with constant agitation, until the optical density at 600 nm (OD_600_) was approximately 0.1, which corresponds to approximately 3.5 × 10^7^ CFU ml^−1^. The cell pellets were harvested by centrifugation at 3500×*g* and subsequently washed in sterile Dulbecco's phosphate-buffered saline (140 mM NaCl, 2.68 mM KCl, 4.23 mM Na_2_HPO_4_, 10 mM KH_2_PO_4_, pH 7.5). The washed cell pellets were finally resuspended in 1 ml of sterile isotonic saline (0.3% (w/v) NaCl). A 100 µl aliquot of the resuspended cells in isotonic saline was dehydrated onto CaF_2_ discs at room temperature for 1 h. *Salmonella enterica* serovar *typhimurium* and *P. aeruginosa* were cultured on nutrient agar (8 g l^−1^ nutrient broth (Merck-Millipore), 5 g l^−1^ NaCl, 12 g l^−1^ agar (Merck-Millipore)) at 37°C overnight.

Dehydrated *P. vulgaris* and *E. coli* cultures were rehydrated in 1 ml of sterile Luria-Bertani broth and prepared in 10-fold serial dilutions. The viability of rehydrated cells was determined by culturing the serial dilutions on 2YT agar (1.6% (w/v) tryptone, 1% (w/v) yeast extract, 0.5% (w/v) NaCl and 1.5% (w/v) agar) at 37°C overnight, aerobically. Following incubation, the number of bacterial colonies, at each dilution, was counted and used to determine the number of colony-forming units per millilitre.

### 4.2. Attenuated total reflection–Fourier transform infrared spectroscopic measurement

For FTIR–attenuated total reflection (ATR) spectroscopy, *S. typhimurium* and *P. aeruginosa* cells were collected by swabbing from an agar culture and directly deposited onto the diamond window of a Golden Gate single bounce micro-ATR module coupled to a Bruker IFS Equinox FTIR spectrometer. The cell populations were dehydrated at room temperature for 1 h before measurement. *Proteus vulgaris* and *E. coli* cells were carefully scratched off the CaF_2_ windows using a clean metal spatula and then deposited onto the ATR diamond. Spectra (50 co-added scans at 4 cm^−1^ resolution) were acquired over the region 4000–600 cm^−1^ and ratioed to a 50-scan background recorded of the diamond window in air. The culture was then gradually rehydrated with phosphate-buffered saline as previously described and spectra were collected systematically during the rehydration [44]. Reference spectra of B- and A-DNA (from calf thymus; Sigma, St. Louis, MO, USA) were collected on the same system with the same parameters as previously described [44]. Spectra were baseline corrected using a concave rubberband method with 12 iterations and 25 baseline points and then vector normalized within experimental sets across the 1435–1140 cm^−1^ region to correct for changes in concentration during dehydration and rehydration. For between species analysis, all spectra were vector normalized together across the 1435–1140 cm^−1^ region. Second derivatives were calculated using nine smoothing points for the Savitsky–Golay process.

### 4.3. Principal component analysis

Baseline corrected and normalized spectra were converted to JCAMP files and imported into the Unscrambler software suite (v. 9.2; CAMO, Oslo, Norway) and collated into one file. PCA was conducted with five principal components over the entire sugar–phosphate backbone fingerprint region (1300–950 cm^−1^) for 12 representative spectra. These 12 spectra were from six cultures (*S. typhimurium*, *P. aeruginosa*, *P. vulgaris* (×2) and *E. coli* (×2)) that were initially dehydrated and then rehydrated.

## Supplementary Material

SI Figure 1

## Supplementary Material

SI Figure 2

## Supplementary Material

SI Figure 3

## Supplementary Material

SI Figure 4
