# Supplementary figures and images for: Detection of an en masse and reversible B- to A-DNA conformational transition in prokaryotes in response to desiccation
Source: J R Soc Interface. 2014 Aug 6;11(97):20140454. doi: 10.1098/rsif.2014.0454 (PMC4208382; doi:10.1098/rsif.2014.0454)

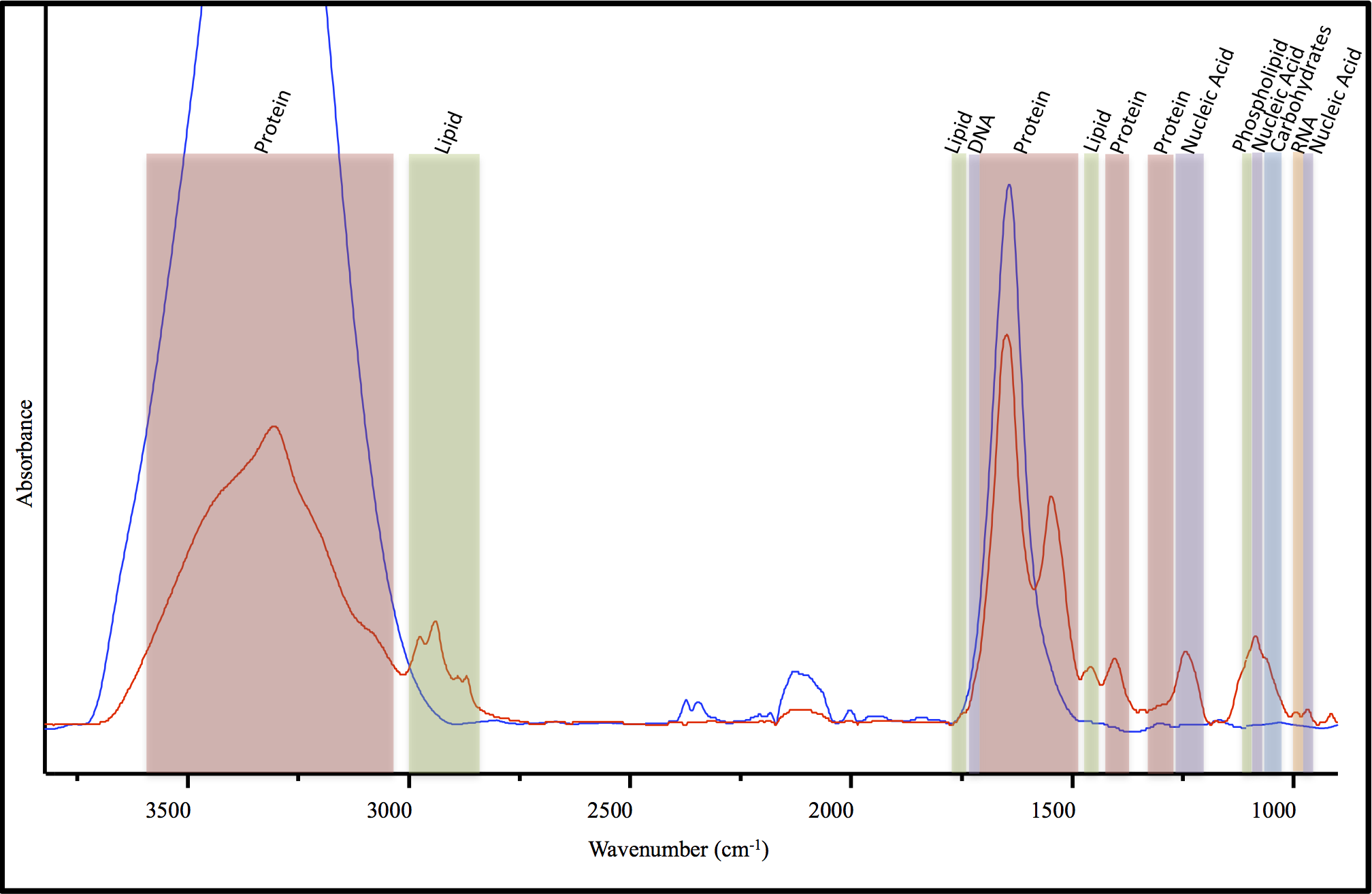

Supplement: SI Figure 1 [file rsif20140454supp1.tiff]

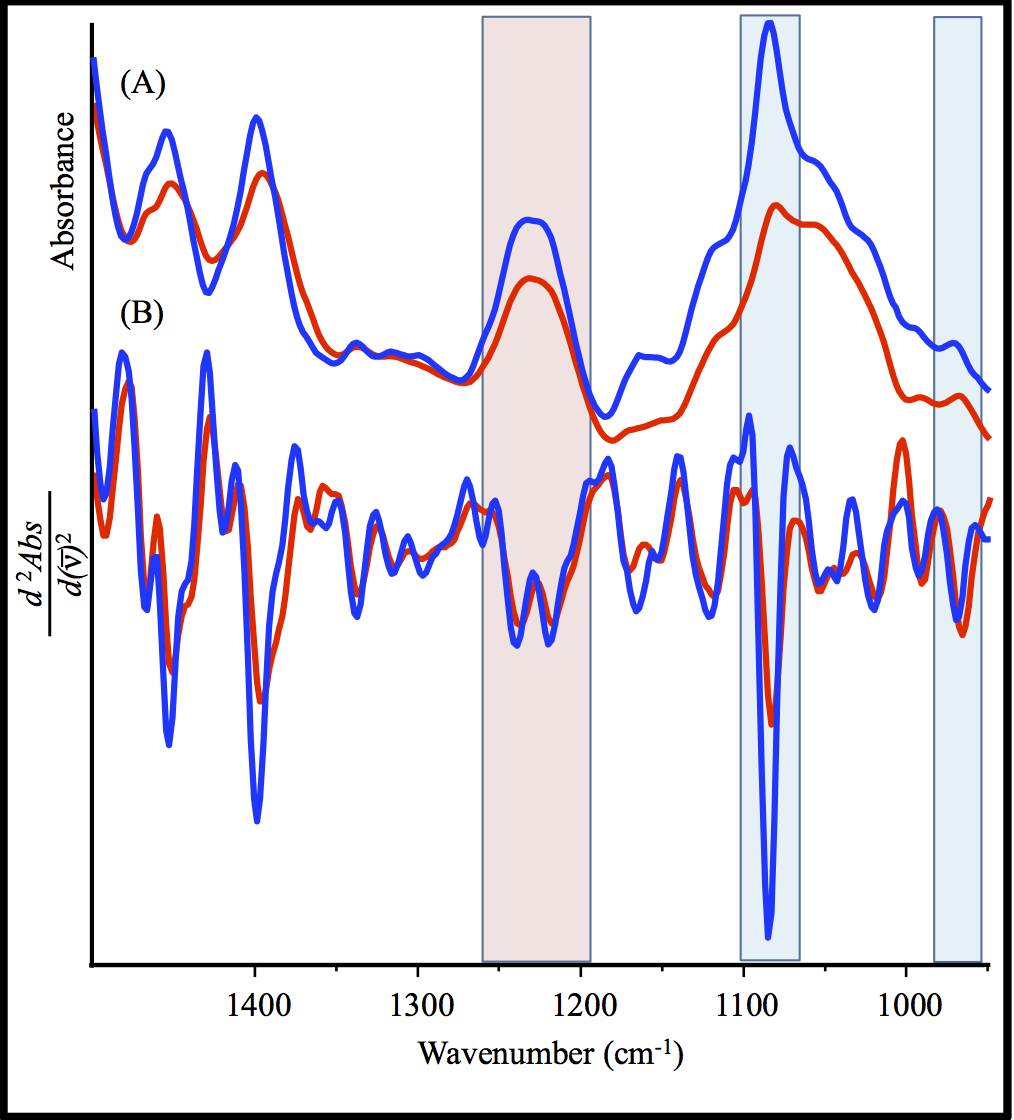

Supplement: SI Figure 2 [file rsif20140454supp2.tiff]

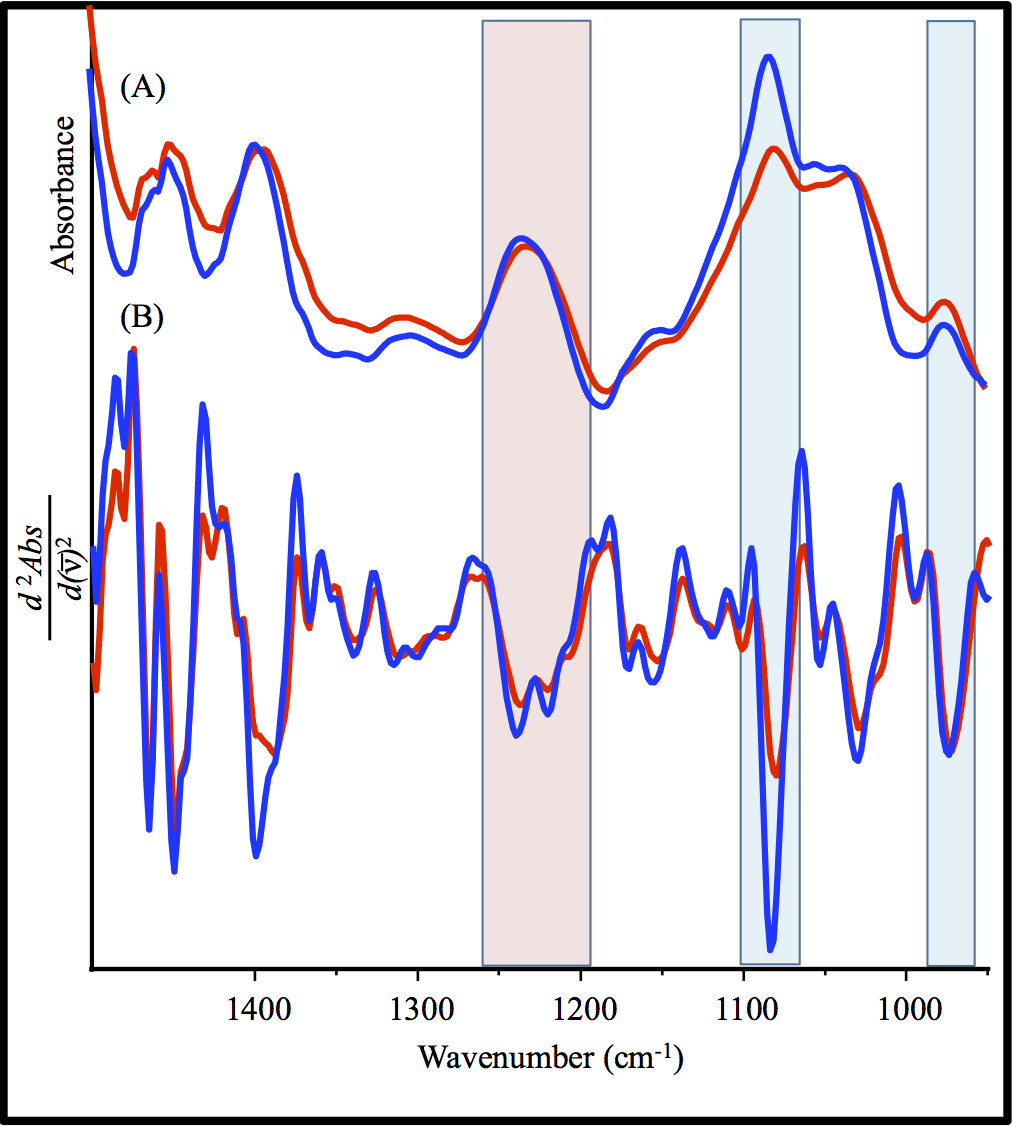

Supplement: SI Figure 3 [file rsif20140454supp3.tiff]

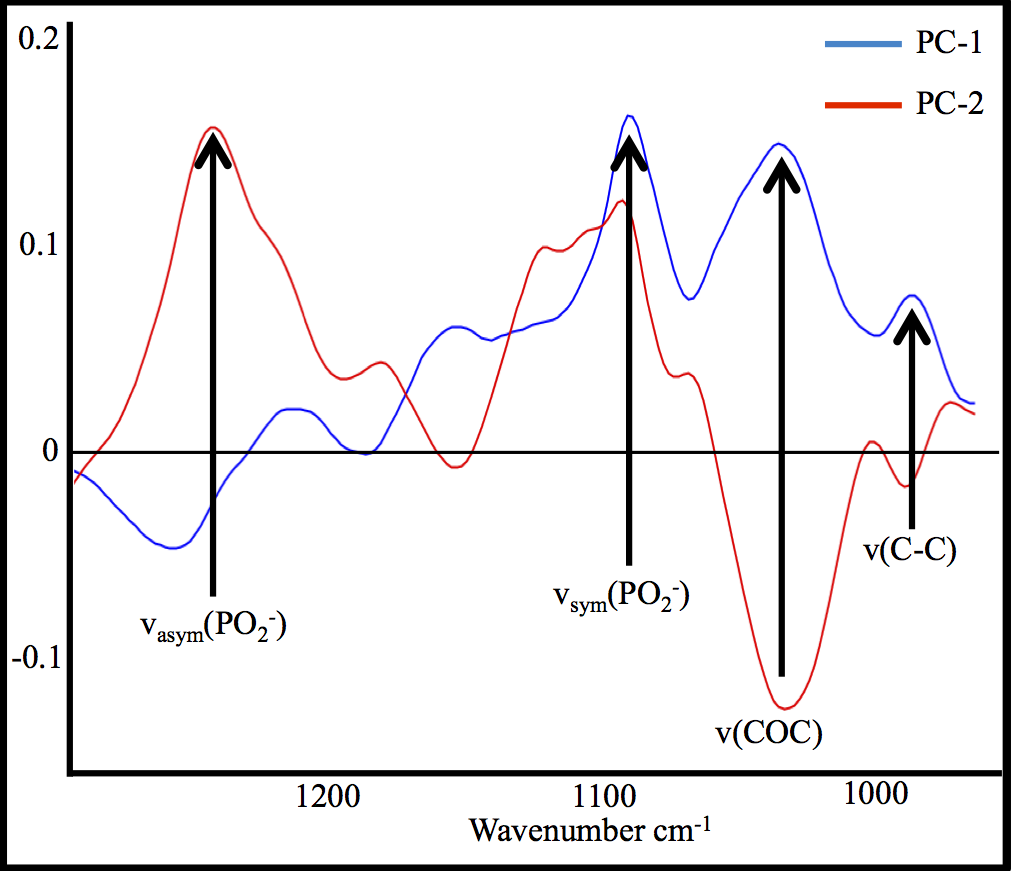

Supplement: SI Figure 4 [file rsif20140454supp4.tiff]
